# Current Progress in Bioactive Ceramic Scaffolds for Bone Repair and Regeneration

**DOI:** 10.3390/ijms15034714

**Published:** 2014-03-18

**Authors:** Chengde Gao, Youwen Deng, Pei Feng, Zhongzheng Mao, Pengjian Li, Bo Yang, Junjie Deng, Yiyuan Cao, Cijun Shuai, Shuping Peng

**Affiliations:** 1 State Key Laboratory of High Performance Complex Manufacturing, Central South University, Changsha 410083, China; E-Mails: gaochengde@gmail.com (C.G.); fengpei@csu.edu.cn (P.F.); maozhongzheng@gmail.com (Z.M.); pengjianli@csu.edu.cn (P.L.); docbo309@csu.edu.cn (B.Y.); dengjunjiejj@163.com (J.D.); csu_caoyiyuan@hotmail.com (Y.C.); 2 Department of Spine Surgery, the Second Xiangya Hospital of Central South University, Changsha 410011, China; E-Mail: drywdeng@163.com; 3 Department of Regenerative Medicine & Cell Biology, Medical University of South Carolina, Charleston, SC 29425, USA; 4 Cancer Research Institute, Central South University, Changsha 410078, China

**Keywords:** bioactive ceramic, nanostructure, pore architecture, preparation technique, bone repair

## Abstract

Bioactive ceramics have received great attention in the past decades owing to their success in stimulating cell proliferation, differentiation and bone tissue regeneration. They can react and form chemical bonds with cells and tissues in human body. This paper provides a comprehensive review of the application of bioactive ceramics for bone repair and regeneration. The review systematically summarizes the types and characters of bioactive ceramics, the fabrication methods for nanostructure and hierarchically porous structure, typical toughness methods for ceramic scaffold and corresponding mechanisms such as fiber toughness, whisker toughness and particle toughness. Moreover, greater insights into the mechanisms of interaction between ceramics and cells are provided, as well as the development of ceramic-based composite materials. The development and challenges of bioactive ceramics are also discussed from the perspective of bone repair and regeneration.

## 1. Introduction

Defects and functional disorder of bone have become a global health care problem [1,2]. Bone repair has become a major clinical and socioeconomic need with the increasing aging population and social development [3,4]. Current medical treatments for bone injuries have been largely focused on replacing the lost bone with allogeneic or autogenous bone grafts, which are limited by many aspects [5]. For example, the amount of donor tissue available and complications at the donor site are limiting factors for autogenous bone grafting. In the case of allogeneic bone grafts, cell-mediated immune responses and pathogen transfer can be problematic [6]. Besides, metal materials, inorganic nonmetallic materials and organic materials have been used for bone repair with the development of medicine, biology and materials science. However, some of them (such as stainless steel, titanium alloy and alumina ceramics) are bioinert materials which limited their clinical applications due to the non-active bond with human tissue [7]. Therefore, bone scaffolds which have the potential to allow new bone tissue ingrowth and mechanical properties to match that of natural bone are in high demand.

Bone tissue engineering (BTE) has been emerging as a valid approach to the current therapies for bone regeneration [8]. In contrast to conventional approach, BTE aims to produce patient-specific biological substitutes in an attempt to circumvent the limitations of existing clinical treatments for damaged tissue or organs. More specifically, BTE typically involves the use of porous and bioresorbable scaffolds to serve as temporary, three-dimensional scaffolds stimulated by growth factors to guide cell attachment, differentiation, proliferation, and subsequent tissue regeneration [9]. The scaffold plays the most important role in BTE because it mimics the function of natural bone [10]. An ideal scaffold should possess the following characteristics [11,12]: (i) good bioactivity, biodegradability, biocompatibility and predictable rate of degradation; (ii) suitable porous structure to promote cell proliferation, vascular ingrowth and nutrient transportation; (iii) suitable surface morphology and physiochemical properties to encourage intracellular signaling; and (v) customized shape to adapt specific damaged bone.

Among various kinds of biomaterials, bioactive ceramics are considered as the most promising material for BTE. Some bioactive ceramics such as hydroxyapatite (HAP), tricalcium phosphate (TCP), bioactive glass (BG) and calcium silicate (CS) have been profusely investigated as biomaterials owing to their capability to form direct bonds with living bone after implantation in bone defects [13]. HAP is a major component and an essential ingredient of natural bone. It can combine with tissues by chemical bonds to form new bone tissue after implanted [14]. TCP has good bioactivity, biodegradability, biocompatibility and it can enhance stem cell proliferation capacity and guide bone regeneration [15]. BG has been investigated as materials for bone repair since the first work of Hench *et al.* at the University of Florida. It can promote gene expression and production of osteocalcin [16]. CS has excellent bioactivity and the ability to bond with living bone and soft tissue. In particular, the Si ion is capable of inducing bone-like apatite formation in simulated body fluid (SBF) [17]. Therefore, bioactive ceramics have become a research hotspot for bone repair and regeneration in the past decades. However, so far, few articles provide comprehensive review of the progress in this field.

This article summarizes the research progress and status of bioactive ceramics scaffolds for BTE at home and abroad. The materials, structure and fabrication technology of ceramic scaffold used in recent years are discussed in detail. The interaction mechanisms between bioactive ceramics and cells are presented. The developments of bioactive nanoceramics and ceramic-based composite materials are also reviewed. Moreover, the problems existed and developing trends of ceramic scaffold for BTE are described.

## 2. Mechanical Properties of Bioactive Ceramics

A scaffold must have appropriate mechanical properties to match and form firm connections with the newly formed tissue due to the complicated stress environment of human skeleton system [18]. High elastic modulus of the scaffold would lead to stress shielding after a long implantation time, and would eventually result in failure of bone repair [19]. The processing condition and application environment largely depend on the physical properties (phase composition, contractibility, porosity and so on) and mechanical properties (compressive strength, elastic modulus, fracture toughness and so on) of the bone scaffold [20].

Bioactive ceramics are known to enhance osteoblast differentiation as well as osteoblast growth. However, their clinical applications have been limited because of their brittleness, difficulty of shaping, and an extremely slow degradation rate in the case of HAP. Also, they have poor fidelity and reliability and new bone formed in a porous ceramic scaffold cannot sustain the mechanical loading needed for weight-bearing bone [21–23].

Wang *et al.* [24] reported that dense HAP ceramics with fracture toughness of 0.61–1.06 MPa·m^1/2^ have been fabricated via conventional sintering. Fielding *et al.* [25] fabricated TCP scaffolds with compressive strength of 1.75–5.48 MPa using commercial three-dimensional (3D) printing technology. However, the mechanical properties of fabricated scaffolds were far below those of weight-bearing bone (fracture toughness: 2–12 MPa·m^1/2^; compressive strength: 130–180 MPa) in the human body [26,27]. Therefore, to obtain an effective method to overcome these limitations has become the focus and focal point of the present and future research in BTE.

## 3. Materials for Strengthening and Toughening

The brittleness of ceramic is attributed to the crack prior to fracture. The control of crack growth prior to fracture can improve the toughness and reduce the stress concentration caused by the acute angle effect of the crack [28]. Therefore, second-phase additions such as fiber, whisker and particles are used to improve the strength and toughness of bioactive ceramics.

### 3.1. Fiber Toughness

Losquadro *et al.* [29] used polylactide-*co*-glycolide (PLGA) fiber as second-phase addition. The results showed that fiber-reinforced calcium phosphate bone cement exhibited more superior structural integrity and material strength than nonreinforced calcium phosphate bone cement. Zhang *et al.* [30] combined suture fibers and chitosan in calcium phosphate cement (CPC). The results showed that CPC achieved substantial synergistic effects. Suping *et al.* [31] investigated the mechanism of interfacial interaction between HAP and carbon fibers. The results revealed that composite reinforced by carbon fibers exhibited significantly improved biomechanical properties.

### 3.2. Whisker Toughness

Whisker is a filament of material that is structured as a single and defect-free crystal. Typical whisker materials are known for having very high tensile strength (on the order of 10–20 GPa) due to the ordered atomic arrangement and periodic structure of lattice [32]. Müller *et al.* [33] reported on the reinforcement of CPC with HAP whiskers. The results showed that the flexural strength of CPC increased by 60% with HAP whiskers (30 vol %). Bose *et al.* [34] used HAP whiskers as reinforcement phase to strengthen HAP. The results showed that satisfactory mechanical and biological properties were obtained. Zhao *et al.* [35] investigated effects of HAP whiskers addition on mechanical properties of CPC. The results showed that the composites obtained good mechanical properties, comparing CPC with non-added whiskers.

### 3.3. Particle Toughness

Ahn *et al.* [36] reported a colloidal addition technique to obtain high dispersion of zirconia (ZrO_2_) nanocrystals within HAP matrix. The results revealed that nanocomposites possessed significantly higher mechanical strength compared with pure HAP and conventional HAP-based composites. Wei *et al.* [37] prepared nano-hydroxyapatite/poly(l-lactic acid) (nano-HAP/PLLA) composite using thermally induced phase separation techniques. The compressive modulus reached 8.3 MPa when the weight ratio of nano-HAP to PLLA was 50:50. Gentile *et al.* [38] prepared cross-linked gelatin/hydroxyapatite/bioactive glass (G/HAP/CEL2) films with different compositions (100:0:0 (G1); 30:70:0 (G2); 30:0:70 (G3); 30:35:35 (G4) (%, *w*/*w*/*w*)) for bone repair. The tensile modulus increased from 4.72 ± 0.23 MPa for G1 to 6.46 ± 0.05 MPa for G4. Recently, they fabricated porous scaffolds made of chitosan/gelatin (POL) blends containing different amounts of CEL2 by freeze-drying [39]. The compressive modulus was up to 2.1 ± 0.1 MPa for CEL2/POL (70/30 *w*/*w*). Zhu *et al.* [40] used *in situ* synthesized ZrO_2_ nanoparticles on the surface of carbon nanotubes as second-phase addition. The results showed that the mechanical properties of alumina ceramics mixed with these composites were much better than that of ceramics alone.

The above methods could improve the strength and toughness of ceramics to some extent. However, some negative effects, including toxicity of whisker and depressed biological properties, are generated by the second-phase additions into matrix [41,42]. In particular, the brittleness of ceramics persisted and could not be thoroughly solved.

## 4. Nanoceramic Artificial Bone

### 4.1. Advantages of Nanoceramic Artificial Bone

Nanotechnology is predominately directing the research of biomedical materials for attaining both sufficient mechanical properties and excellent biological performances [43]. It is well known that nano-materials have superior strength and toughness that significantly exceed the corresponding characteristics of conventional materials. This may because that the bubbles and defects in materials decrease with the size of crystalline grain into nanometer [44]. Moreover, with a decrease in grain size, the effect of conventional lattice dislocation slip on fracture toughness enhancement significantly decreases. At the same time, fracture toughness can be improved due to the increased grain boundary sliding and migration [45]. Nano-materials also have excellent torsion modulus and tensile modulus, which is ascribed to the small grain size, large surface area and strong interfacial interactions [46]. Therefore, nanoceramics are promising to fundamentally solve the brittleness of bioactive ceramics.

Besides, the scaffold fabricated with nanoceramics also has superior properties in other respects. For example, scaffold degradation could match the growth rate of new bone by controlling the size of crystalline grain, which is important to improve bone graft healing rate and reduce complications. Researchers also found that nano-HAP can significantly suppress the growth of cancer cell line while have no effect on normal cells [47]. There are nanoscale holes, fibers and protuberances in the nature extracellular matrix (ECM) due to its 3D nanofiber structure. In addition, the cell surface receptor has a nanostructure and the size of structural-functional domains on cell surfaces is also nanoscale. Moreover, the interactions among biological molecules, the components of ECM and cells all occur on the nanoscale, which has direct influence on the behaviors and functions of cells (such as cell proliferation and gene expression) [48–50]. It is thus clear that the scaffold with nanostructure can imitate well the structure and biological function of the nature of ECM and achieve full integration with the body tissue. Therefore, there is an urgent need to manufacture a novel scaffold with specific structure and function by nanotechnology in tissue engineering.

### 4.2. Processing Methods

It is known that nano-materials have tremendously specific surface area, high surface energy and that their atoms lack coordination [51]. It is difficult to obtain nanostructure during the fabrication of scaffold. Many studies said that surface diffusion plays a dominant role and leads to grain coarsening at the early stage of conventional furnace sintering. Moreover, it needs several hours or more of heat preservation for the densification process, during which the grains would grow from nanoscale to microscale [52]. Researchers have carried out many studies to restrain grain growth during fabrication of scaffold, such as utilizing additives and new processing technology. Among these methods, adopting new processing technology is preferred since it will not introduce a second phase.

In order to restrain grain growth during fabrication of scaffold, many methods have been adopted to accelerate the sintering process. Lots of researchers tried to fabricate nanoceramics by hot pressing sintering or vacuum sintering [53–55]. However, the products present nonuniform microstructures which have bad effects on the mechanical properties of scaffold. In addition, the cost of these methods is very high. Recently, new sintering processes such as microwave sintering and spark plasma sintering have attracted great interest because they have high heating rate and are able to improve homogeneity and properties of the materials [56–58]. However, the types of materials are extremely limited in microwave sintering and there is coarse-grained local in spark plasma sintering [59,60]. Moreover, it is difficult to manufacture customized implants because their shapes are limited by the sintering mold.

Selective laser sintering (SLS) method is one of widely used rapid prototype technology [61]. It is able to quickly manufacture customized implants with complicated internal structure based on computer-aided design (CAD) data [62]. The scaffold is fabricated via layer-by-layer process without a mold. More importantly, it is able to restrain the grain growth of nanoceramics by quick heating and sintering process [63]. Nowadays, related studies mainly focus on the polymer or polymer/ceramic composites. Williams *et al.* [64] fabricated poly(ɛ-caprolactone) (PCL) scaffold via SLS, then they investigated its microstructure, mechanical and biological properties. Liao *et al.* [65] fabricated PCL/β-TCP scaffold and investigated its mechanical strength.

## 5. Porous Structure and Corresponding Preparation Technique

Besides the proper mechanical properties, it is a key point to obtain porous structure to create a microenvironment for cell adhesion and proliferation. Nature bone has multi-level three-dimensional pore structure which ranges from several nanometers to several hundreds of micrometers [66]. It can satisfy different requirements of tissue growth. Pores in the range of 150–800 μm allow the ingrowth of bone tissue and blood vessels. Pores in the range of 10–100 μm are beneficial for the growth of blood capillaries, exchange of nutrients and excretion of waste products. Pores in the range of nanoscale provide larger specific surface area and more active targets, which is good for the formation of apatite and the attachment of protein or osteoblast [67]. Meanwhile, it is also important for the adjustment of cell adhesion and proliferates.

### 5.1. Conventional Preparation Methods

Conventional methods for the fabrication of porous structure mainly include pore-forming method, sintered microsphere method and chemical foaming method, *etc*. [68]. The pore-forming method is adding pore-forming agent into ceramic and then burning out to leave pores. The sintered microsphere method is adding polymer microsphere into the mold then heating to glass transition temperature, heat preservation, cool and demold to obtain porous scaffold. The chemical foaming method is mixing chemical foaming agent with ceramic and undergoing pyrolysis to produce gas that are used to foam the ceramic. Li *et al.* [69] fabricated bioglass scaffold with 50 μm macropores and 2–6 nm mesopores via the pore-forming method. Qing *et al.* [70] fabricated PLGA/nano-HAP composite scaffold with 200 μm pores via the sintered microsphere method. Juillerat *et al.* [71] fabricated foam scaffold with 30 μm–1 mm pores via the chemical foaming method. However, these methods have poor controllability over pore structure (such as pore size and connectivity) and shape. The fabricated scaffold is still hugely different compared to the nature of bone pore structure and shape.

### 5.2. Rapid Prototype Technology

Several methods have been applied successfully for the fabrication of scaffold with controlled pore structure and shape, such as fused deposition modeling (FDM), stereo lithography appearance (SLA), SLS and 3D printing technology. Espalin *et al.* [72] investigated the use of medical-grade polymethylmethacrylate (PMMA) in FDM to fabricate porous customized freeform structures. Simon *et al.* [73] fabricated HAP scaffolds with 3D periodic quadrant architectures by direct-write assembly of concentrated colloidal HAP ink. Maeda *et al.* [74] fabricated HAP/Acrylic resin scaffold with uniform pore sizes and regular shape via SLA. Simpson *et al.* [75] investigated PLGA/HAP and PLGA/TCP for the role of a porous scaffold using SLS fabrication process with powder sizes of 50–125 and 125–250 μm. Wu *et al.* [76] prepared highly uniform CS scaffolds with controllable pore structure and improved mechanical strength by applying a modified 3D-printing method. Bian *et al.* [77] designed and fabricated a novel vascularization core implant with porous structure and pre-set channels from β-TCP powder by ceramic stereo lithography. Lin *et al.* [78] designed and prepared bone tissue engineering scaffolds with gradient controllable structure of both macro and micro pores by integrating rapid prototyping and freeze drying technology. Duan *et al.* [79] successfully fabricated three-dimensional nanocomposite scaffolds based on CHAP and PLLA using SLS. The sintered scaffolds had controlled material microstructure, totally interconnected porous structure and high porosity. However, using rapid prototype technology, it is difficult to fabricate scaffold with pores ranging from several nanometers to several microns due to limitation of spot diameter or muzzles diameter. Thus, it could not realize the requirements for the growth of different tissues.

## 6. The Interaction between Bioactive Ceramic Scaffold and Cells

Bone repair and regeneration are a complex interaction processes among scaffolds, cells and microenvironment [80]. The surface property, pore structure and grain size of scaffold are important to cell proliferation, osteoblast activity and vasculogenesis. With the development of molecular biology and bone tissue engineering, current studies mainly focused on preparing bioceramics with specific components and structure aiming to stimulating special cellular response at the molecular level [81]. Yeung *et al.* [82] investigated stem cell therapy by tissue engineering method using TCP ceramics in rabbit model. The osteogenic cells were seeded on TCP ceramics for one day, followed by the implantation of cell-ceramics composite. Results showed that the mesenchymal stem cells (MSCs) derived osteogenic cells augmented spinal fusion and bone mineralization. Jo *et al.* [83] fabricated a PCL/BG nanocomposite using BG nanofibers (BGNFs). *In vitro* cell tests using the MC3T3 cell line demonstrated excellent biocompatibility and high level of bioactivity. *In vivo* animal experiments using Sprague-Dawley albino rats revealed good bone regeneration capability of PCL/BGNF composite when implanted in a calvarial bone defect. Xu *et al.* [84] investigated *in vivo* bone-regenerative capacity and resorption of porous β-calcium silicate (β-CS) bioactive ceramics. The ceramics were implanted in rabbit calvarial defects and harvested after 4, 8 and 16 weeks. Results suggested a cell-mediated process involved in the degradation of β-CS *in vivo.* Silva *et al.* [85] evaluated the ability of macroporous CPC scaffolds and reported that the scaffolds enables adhesion, proliferation, and differentiation of MSCs after 10–15 days of culture in osteogenic cells. McCullen *et al.* [86] seeded human adipose-derived stem cells onto electrospun composite scaffolds and found that they were able to induce osteogenic proliferation and differentiation. Xu *et al.* [87] cultured MSCs on a novel bioactive glass-collagen-hyaluronic acid-Phosphatidylserine composite scaffold for 2 weeks and then implanted them into a rat bone defect model. The results revealed that the introduction of MSCs enhanced the efficiency of new bone formation. In addition, the ionic products released from bioactive ceramic can promote cell proliferation, differentiation and gene expression. Some silicate ceramics showed poor biocompatibility because their fast dissolution rate. This resulted in a rapid increase of pH in cell culture medium thus inhibiting cell growth [88].

## 7. Ceramic-Based Composite Scaffolds

Natural bone is a connective tissue largely composed of organic protein, collagen, inorganic mineral and cells [89]. Obviously, there are congenital deficiencies for a single material to mimic the composition of natural bone. Therefore, researchers started to develop composite materials to meet various properties of scaffolds. It is likely that a high bionics bone matrix would be obtained by taking advantage of the respective virtue of composite materials.

### 7.1. Composites with Natural Biomaterials

Natural biomaterials such as collagen, chitin, coral and chitosan demonstrate good biocompatibility and cellular affinity [90]. Not only can their degradation products be completely absorbed by the body, but also they have the advantage of low price and broad source. However, their durability and strength are insufficient and degradation speeds are uncertain. Yoon *et al.* [91] prepared HAP/chitosan-alginate composite scaffolds through *in situ* co-precipitation and investigated the porosity, morphology, microstructure and mechanical properties of the scaffolds. The results showed that the introduction of HAP improved the compressive strength of scaffold and the pore size gradually decreased with the increase of HAP content. Cheng-zhen *et al.* [92] produced a nano-HAP/collagen composite scaffold. *In vivo* implantation in rats showed no inflammatory response after several weeks, and nano-HAP/collagen composite scaffold was found to promote new blood vessels formation and bone regeneration. Jingushi *et al.* [93] inoculated bone morphogenetic protein-2 into the hip abductor muscle of rabbits with beta-tricalcium phosphate and found it could induce the formation of intramuscular bone with rich vascularity.

### 7.2. Composites with Metallic Materials

Metallic materials (including stainless steel, titanium (Ti), Co-Cr-Mo alloy and titanium-nickel alloy) have become the material of choice for load-bearing implant applications due to high strength, good fatigue resistance and well machining properties. Among these materials, Ti is bioinert, resistant to corrosion and biocompatible with bone tissue. However, some metallic materials may produce adverse effects such as the release of significant amounts of metal ions into the tissues, which may result in complications such as inflammatory and immune reactions [94]. Thus, there is a need to further improve the biocompatibility between metallic materials and host bone. Wang *et al.* [95] prepared porous TiNbZr alloy scaffold by space-holder sintering and calcium phosphate coating is applied to the surface of the scaffold to improve osteoconductivity. Cell culture experiments showed that the porous TiNbZr scaffold was more favorable for osteoblast-like cell adhesion and proliferation. Xiong *et al*. [96] found that *in vitro* proliferation of the osteoblast-like cells was significantly enhanced on the nano-hydroxyapatite coated titanium-niobium alloy compared to the titanium-niobium alloy without coating.

### 7.3. Composites with Biodegradable Polymers

Biodegradable polymers are widely used as biomaterials for the fabrication of cartilage tissue engineering scaffolds. Besides, polymers have great design flexibility because their composition and structure can be tailored to specific needs [97]. Biodegradable polymers, including poly(lactic acid) (PLA), poly(glycolic acid) (PGA) and their copolymers (PLGA), are the most frequently used biodegradable polymer materials. They offer distinct advantages of good biocompatibility, biodegradation, absorbability and high toughness. Unfortunately, these polymers exhibit some drawbacks, such as poor cell affinity and cell-matrix interaction. In addition, polymers will degrade and release acidic degradation products, which are likely to trigger aseptic inflammation reaction and swelling [98,99]. Cao *et al.* [100] fabricated three-dimensional porous composite scaffolds of PGA/β-TCP (in 1:1 and 1:3 weight ratios) by a combination of the solvent casting and particulate leaching method. Then the scaffolds were implanted in rat and characterized by quantitative imageology analysis and qualitative histological evaluations. They observed that the bone began to reform within 14 days and healed well at 30 days after surgery. By 90 days, the bone replacement was almost completed and presented a healthy bone appearance. The PGA/β-TCP scaffold with a weight ratio 1:3 exhibited a strong ability for osteogenesis, mineralization and biodegradation. Huang *et al.* [101] fabricated PLGA/nano-HAP composite scaffolds by thermally induced phase separation technique and investigated the effects of solvent composition, polymer concentration, coarsening temperature, and coarsening time as well as nano-HAP content on the micro-morphology, mechanical properties of the scaffolds. They proved that the introduction of nano-HAP greatly increased the mechanical properties and water absorption ability. Meanwhile, the PLGA/nano-HAP scaffolds also exhibited significantly higher cell growth and alkaline phosphatase (ALP) activity.

### 7.4. Composites of Two or More Bioactive Ceramics

Bioactive ceramics differ from each other in mechanical properties and biological properties. A combination of two or more bioactive ceramics will provide a wide space for ceramic material design. Moreover, multiphase ceramic is expected to remedy the drawback of some materials and reach a better comprehensive performance [102]. A single phase ceramic material has achieved considerable improvement in mechanical properties by incorporating one or more other components to form ceramic matrix composites. Lee *et al.* [103] fabricated and evaluated porous β-TCP/HAP (60/40 *w*/*w*) composite as a bone graft extenders. The results showed the composite increased new bone formation in the calvarial defect model both quantitatively and qualitatively. Lin *et al.* [104] fabricated HAP/CS composite bioceramics with different weight ratio and investigated effects of composite ratio on bioactivity, degradability behavior and MSCs response to the composites. It was concluded that the degradability of the HAP/CS composite bioceramics could be tailored by adjusting the initial HAP/CS ratio. In addition, the proliferation rate of MSCs was significantly higher than that of pure HAP. Hesaraki *et al.* [105] developed β-TCP/BG composites and they found abundant precipitation of calcium phosphate layer on the surfaces of the β-TCP/BG composites after soaking in SBF. The β-TCP/BG composites exhibited better growth of human osteoblastic cells than that of pure β-TCP.

## 8. Novel Bioactive Ceramic Scaffold with Nano Grain and Hierarchically Porous Structure

**Figure 1.**
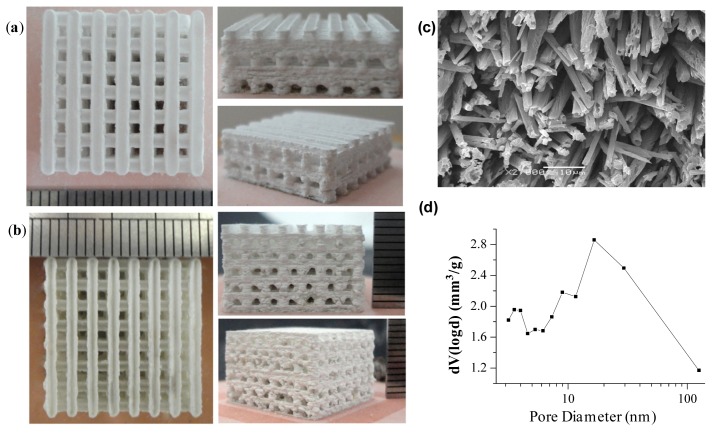
(**a**,**b**) Bioactive ceramic (TCP) scaffolds fabricated by SLS; (**c**) Micro/nanometer-sized porous morphology; and (**d**) Pore size distribution curve on the etched surface of HAP/TCP (60/40 *w*/*w*) ceramic scaffold.

An ideal scaffold should be biocompatible, bioactive and biodegradable for osteogenesis, osteoinduction and osteoconduction. In addition, the scaffold should possess sufficient porosity to accommodate cell proliferation and differentiation. It is also desirable for scaffolds to have adequate mechanical property and appearance in accordance with the defect parts. Our research team developed a homemade selective laser sintering system in order to prepare nano-ceramic artificial bone with outstanding mechanical properties. The minimum spot diameter of laser can reach 50 μm with the focusing system. The SLS system could realize arbitrary complex movements based on the non-uniform rational B-Spline (NURBS) theory [106,107]. We have fabricated a variety of ceramic scaffolds using the system. It is able to prepare interconnecting porous structure (pore sizes: hundreds of micrometers) through adjustment of laser parameters and scanning space (Figure 1a,b). Multilayer stereo micro/nanometer-sized porous surface structures can be prepared by selective chemical etching of biphasic ceramic scaffolds in phosphoric acid solution (Figure 1c). The nitrogen adsorption data indicated hierarchical pore size distribution at 3–5 and 10–100 nm, as well as pore size above 100 nm on the scaffold surface (Figure 1d).

**Figure 2.**
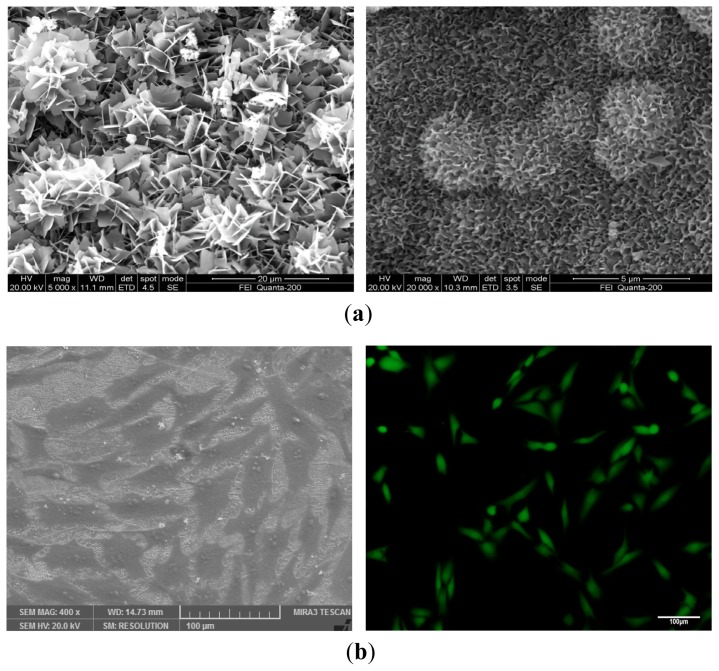
(**a**) Surface morphology of TCP ceramic scaffold after immersing in SBF; (**b**) MG-63 cells on TCP ceramic scaffold doped with 2.5 wt % ZnO.

**Figure 3.**
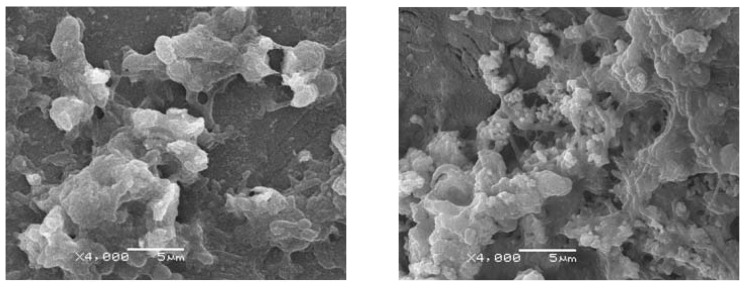
Human bone marrow mesenchymal stem cells (hBMSCs) differentiate to osteoblast on the TCP ceramic scaffold.

An *in vitro* experiment in SBF indicates the gradual degradation of scaffold with the increase of immersing time (Figure 2a). A layer of bone-like apatite forms on the scaffold surface as the scaffold degrades. The bone-like apatite morphology evolves from flake-like to sponge-like and grows from the scaffold surface to the whole scaffolds [108,109]. Biphasic calcium phosphates (BCP) with different concentrations of HAP and β-TCP are found to possess quite a different degradation rate [110]. MG-63 cells are seeded on the scaffolds to evaluate cell-scaffold interaction. The results show that MG-63 cells could attach and proliferate well on the scaffolds, indicating good biocompatibility of the scaffolds (Figure 2b). Human bone marrow mesenchymal stem cells (hBMSCs) are used to investigate the ability of ceramic scaffold to induce osteogenesis (Figure 3). The high levels of ALP and calcium deposits indicate successful induction of hBMSCs’ differentiation into osteoblasts. Besides, the number of adherent cells on scaffold surface increases with the increasing of micro/nanometer-sized pores on the scaffold surface, which provide location for cell adhesion and migration and facilitate the formation of gap junction between cells. In addition, incorporation of Mg, Zn and Si elements in bioactive ceramics may be helpful for regulating the physicochemical and biological properties. Based on the preliminary study of our research team, it is feasible to fabricate nanoceramic bone scaffold with sufficient mechanical properties, hierarchically porous structure, 3D shape and controllable degradation by choosing appropriate materials and processing parameters.

## 9. Conclusions and Perspectives

The development of BTE provides an effective approach for bone repair and regeneration. The selection of scaffold material and structure optimization is quite important in order to fully mimic the 3D network structure of bone ECM. Among various kinds of biomaterials, bioactive ceramics are drawing more and more attention due to excellent biocompatibility, degradability and osteogenesis. Furthermore, bioactive ceramics possess the ability to induce differentiation of various stem cells. This paper summarizes the latest research progress of bioactive ceramics as bone scaffold materials, including main types of bioactive ceramics, mechanical properties, strengthening and toughening methods, bone-like apatite mineralization ability and cellular biocompatibility. The wide range of chemical composition of bioactive ceramics provides superior material foundation for control and optimization of the physicochemical and biological properties. Nanotechnology and composite materials provide new ways to improve the strength and toughness of bioactive ceramics, which further sophisticates the physicochemical and mechanical properties for BTE applications. Moreover, bioactive nanoceramics are exhibiting great potential for bone repair than conventional ceramics due to better mechanical and biological properties. Future studies should be focused on the responding mechanism between bioactive nanoceramics and cells on molecular and genetic level. On the other hand, *in vivo* experiments are necessary to master the evolution mechanism of mechanical properties and degradation of bioactive nanoceramics. We believe bioactive nanoceramics will become an ideal bone substitute material that is widely used in bone repair and regeneration in the future.
